# Pathological investigation of high pathogenicity avian influenza H5N8 in captive houbara bustards (*Chlamydotis undulata*), the United Arab Emirates 2020

**DOI:** 10.1038/s41598-024-54884-2

**Published:** 2024-02-20

**Authors:** Manuela Crispo, Mar Carrasco Muñoz, Frédéric Lacroix, Mohamed-Reda Kheyi, Maxence Delverdier, Guillaume Croville, Malorie Dirat, Nicolas Gaide, Jean Luc Guerin, Guillaume Le Loc’h

**Affiliations:** 1 IHAP, Université de Toulouse, ENVT, INRAE, 23 Chemin des Capelles, 31076 Toulouse Cedex 3, France; 2 Reneco International Wildlife Consultants LLC, PO Box 61741, Abu Dhabi, United Arab Emirates

**Keywords:** Influenza A virus, H5N8 subtype, Endangered species, United Arab Emirates, Immunohistochemistry, Nanopore sequencing, Molecular biology, Infectious diseases, Viral infection

## Abstract

At the end of 2020, an outbreak of HPAI H5N8 was registered in captive African houbara bustards (*Chlamydotis undulata*) in the United Arab Emirates. In order to better understand the pathobiology of this viral infection in bustards, a comprehensive pathological characterization was performed. A total of six birds were selected for necropsy, histopathology, immunohistochemistry, RNAscope in situ hybridization and RT-qPCR and nanopore sequencing on formalin-fixed and paraffin-embedded (FFPE) tissue blocks. Gross lesions included mottled and/or hemorrhagic pancreas, spleen and liver and fibrinous deposits on air sacs and intestine. Necrotizing pancreatitis, splenitis and concurrent vasculitis, hepatitis and fibrino-heterophilic peritonitis were identified, microscopically. Viral antigens (nucleoprotein) and RNAs (matrix gene) were both detected within necro-inflammatory foci, parenchymal cells, stromal cells and endothelial cells of affected organs, including the myenteric plexus. Molecular analysis of FFPE blocks successfully detected HPAI H5N8, further confirming its involvement in the lesions observed. In conclusion, HPAI H5N8 in African houbara bustards results in hyperacute/acute forms exhibiting marked pantropism, endotheliotropism and neurotropism. In addition, our findings support the use of FFPE tissues for molecular studies of poorly characterized pathogens in exotic and endangered species, when availability of samples is limited.

## Introduction

The concept that high pathogenicity avian influenza (HPAI) outbreaks were confined mostly to poultry flocks, as a result of the introduction of low pathogenicity viral subtypes H5 and H7 originating from wild aquatic birds, was shattered by the emergence of the A/goose/Guangdong/1/1996(Gs/Gd) H5N1 influenza A virus^1,2^. Since 2002, mortality events associated with Gs/Gd HPAI viruses, especially from clade 2.3.4.4b, have been reported in an variety of avian species, including endangered ones^1^. A few recent examples include the demise of more than 6500 Cape cormorants (*Phalacrocorax capensis*) and 350 African penguins (*Spheniscus demersus*) in Namibia, the loss of 750 great white pelicans (*Pelecanus onocrotalus*) belonging to the Djoudj National Bird Sanctuary, near the Senegal–Mauritania border and massive die-offs involving breeding populations of great skuas (*Stercorarius skua*) in Great Britain^3–7^.

Numerous avian species are the object of captive breeding and reinforcement programs, actively engaged in preserving and restoring wild populations. From this point, a remarkable example is provided by the African houbara bustard (*Chlamydotis undulata*) a terrestrial bird inhabiting the semi-desertic regions of North Africa and Canary Islands, classified as vulnerable by the International Union for the Conservation of Nature^8^. As a result, several conservation breeding projects have been established in North Africa and United Arab Emirates. Infectious diseases represent a significant threat for captive-breeding and release programs, potentially impairing repopulation efforts on multiple levels: loss of valuable individuals, including breeding adults, reduced breeding success and survival rates, local spread of harmful pathogens following the exposure of native wild populations to harmful pathogens associated with the translocation of infected individuals for restocking purposes^9–12^. Predator and scavenger species may also be threatened, due to the consumption of infected animals^13–15^. Additional risks arise from the rehabilitation of illegally traded birds into the wild or their introduction within captive breeding projects^16^. Quarantine measures, routine health monitoring and screening, are essential to properly understand sanitary issues and implement the most effective control measures, especially in species for which the presentation of a variety of pathogens is still poorly described^16^. From this point of view, necropsy findings can provide useful insights, guiding the diagnostic process, through target sampling^17–19^.

The clinico-pathological picture of HPAI can vary significantly, due to both virus-related and host-related factor^20,21^. While our knowledge on the pathobiological features of HPAI infection in captive and, to a lesser extent, wild bird species have been expanded by a variety of research works, available literature on bustards is still limited^7,13,15,18,21–24^.

To the authors’ best knowledge, this is the first pathological description of H5N8 HPAI natural infection in captive African houbara bustards in the United Arab Emirates, including an overview of viral tissue distribution based on two different in situ detection techniques and the use of formalin-fixed and paraffin-embedded (FFPE) tissue samples for molecular analysis.

## Materials and methods

### Selection of birds belonging to a H5N8 HPAI outbreak

In November 2020, an outbreak of H5N8 HPAI was registered in a flock of captive African houbara bustards, part of a breeding conservation project established in the United Arab Emirates (International Fund for Houbara Conservation)^25^ Affected birds, all vaccinated against H5N2 (inactivated vaccine) 4.5 months before, ranged between 18 to 32 months of age (average 22 months) and were housed in outdoor pens, including 4–7 bird each, for a total of 314 individuals. Starting from November 25th, over a 7 days period, 8 birds belonging to 6 different pens died. Of these, 6 were found dead without exhibiting clinical signs, while 2 appeared mildly lethargic 24–48 h prior to the *exitus*. An additional bird exhibited similar signs for 24 h. Due to the drastic decline of its conditions, a decision was made by the veterinarian and the bird was humanely euthanized by intravenous injection of T-61.

H5N8 HPAI infection was confirmed by real-time quantitative reverse transcription polymerase chain reaction (RT-qPCR) targeting the subtype H5 of hemagglutinin (HA) and by HA and neuraminidase (NA) sequencing on swabs (oropharyngeal and cloacal) and fresh tissue sections (lung and spleen). Among the eight birds that died spontaneously, six birds were randomly selected for pathological investigation, including four birds experiencing hyperacute death (three males and one female) and two symptomatic individuals (one male and one female).

### Pathology and viral tissue distribution

A complete necropsy was performed within 48 h after the *exitus.* For histopathology, sections of trachea, lung, heart, liver, spleen, pancreas, gizzard, small and large intestine, cecal tonsils, kidney and ovary were fixed in 10% neutral buffered formalin, paraffin-embedded and sectioned at 3 µm. Slides were stained with hematoxylin and eosin (H&E) and examined by light microscopy. Necrotic-inflammatory lesions were scored according to severity^26^.

For viral tissue distribution, serial sections, 3 µm-thick, were obtained FFPE tissues, mounted on charged slides and stained with immunohistochemistry (IHC) and RNAscope in situ hybridization (ISH). The immunohistochemical assay was performed using a monoclonal mouse antibody directed against influenza A virus (IAV) nucleoprotein (NP) (Clone HB65, FCG013, Kerafast, Boston). Sections of IAV positive tissues were used as positive controls. Negative controls included sections incubated without the primary antibody or with another monoclonal antibody belonging to the same isotype (IgG2). The RNAscope ISH assay relied on a custom-designed probe (V-H5N8-M1M2) targeting the well-conserved influenza matrix protein genes (M gene) 1 and 2 of HPAIV (H5N8) Clade 2.3.4.4b (A/chicken/France/20P016448/2020) following the RNAscope® 2.5 HD Assay RED (Advanced Cell Diagnostics, Hayward, CA)^27,28^. A probe targeting the dihydrodipicolinate reductase gene from the *Bacillus subtilis* strain SMY, served as negative control, while sections of AIV positive tissues were used as positive controls. Parenchymal and endothelial immunoreactivity of each organ were scored according to distribution for both techniques^26^.

### Molecular analysis on FFPE tissue blocks

A total of 4 FFPE tissue blocks, obtained from 3 birds and including sections of liver, spleen, pancreas and intestine exhibiting necrotic-inflammatory lesions and positive immunostaining, were selected for molecular analysis. RNA was extracted using the FormaPure XL RNA Kit (Beckman Coulter, Indianapolis, IN, USA), following manufacturer’s recommendations. Qualitative and quantitative analysis of extracted RNA and DV200 assessment were conducted using high-throughput, automated electrophoresis (Tapestation 4200, Agilent). Subsequently, RT-qPCR targeting AIV H5 was performed (Influenza H5/H7 ID Gene PCR kit, IDVet), followed by HA and NA sequencing using the MinION Mk1C device (Oxford Nanopore Technologies, Oxford, UK) according to the protocol detailed by Croville et al*.*^29^.

## Results

### Gross pathology

**Figure 1:**
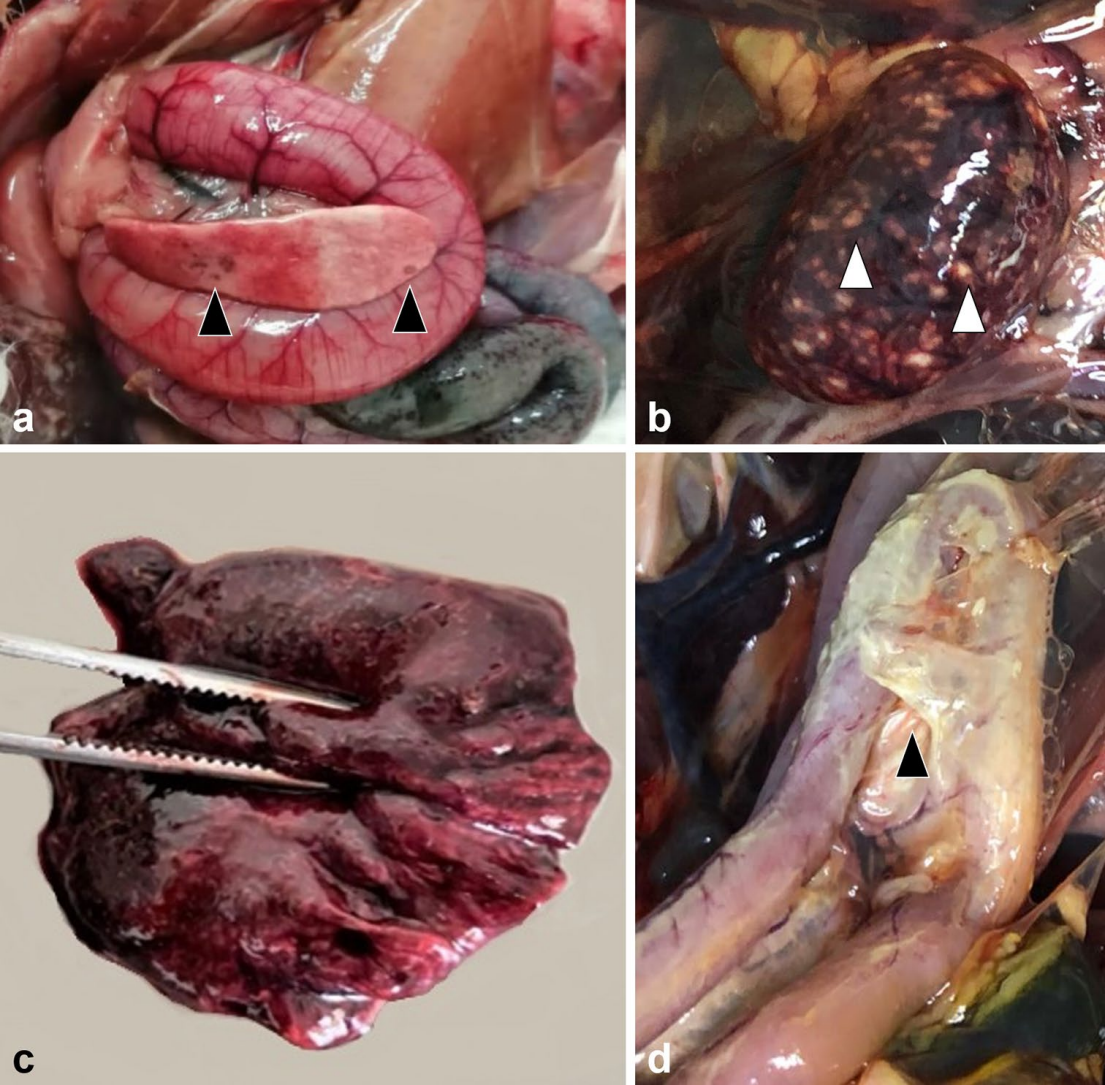
Necropsy findings in African houbara bustards (*Chlamydotis undulata*) naturally-infected with H5N8 HPAIV. Mottled pancreas with multiple hemorrhagic foci (arrowheads) (**a**). Diffusely enlarged and mottled spleen with multiple necrotic foci (arrowheads) (**b**). Diffusely congested and edematous lungs (**c**). Accumulation of yellow fibrinous exudate on the surface of the small intestine (duodenum) (**d**).

Selected birds were mostly in good nutritional status (5/6 birds). The pancreas was diffusely enlarged, firm and mottled (4/6) or hemorrhagic (2/6) (Fig. 1a). The liver was diffusely enlarged, pale and firm (5/6) or friable (1/6). The spleen was enlarged and mottled with a gritty consistency (5/6) or hemorrhagic (1/6) (Fig. 1b). Multiple, pale foci, 1 to 3 mm (mm) in diameter, were visible on the surface of liver (5/6) and spleen (3/6). Kidneys were diffusely congested (4/6) or pale and swollen (2/6). The glottis (2/6), tracheal mucosa (4/6) and lungs (3/6) were markedly congested and edematous (Fig. 1c), while white fibrinous plaques were present in the oropharynx (3/6). Yellow fibrinous exudate was covering the thoracic and abdominal air sacs, extending to the surface of the small intestine and pancreas (3/6) (Fig. 1d). Multifocal to coalescing petechial hemorrhages were identified in the ovary (1/6), gizzard’s mucosa and underneath the koilin (1/6), the mucosa of the small intestine and cecal tonsils (2/6). Occasional ulcers were noticed in the cecal tonsils and colon mucosa (1/6). Mild hydropericardium was present in one bird.

### Histopathology

**Figure 2:**
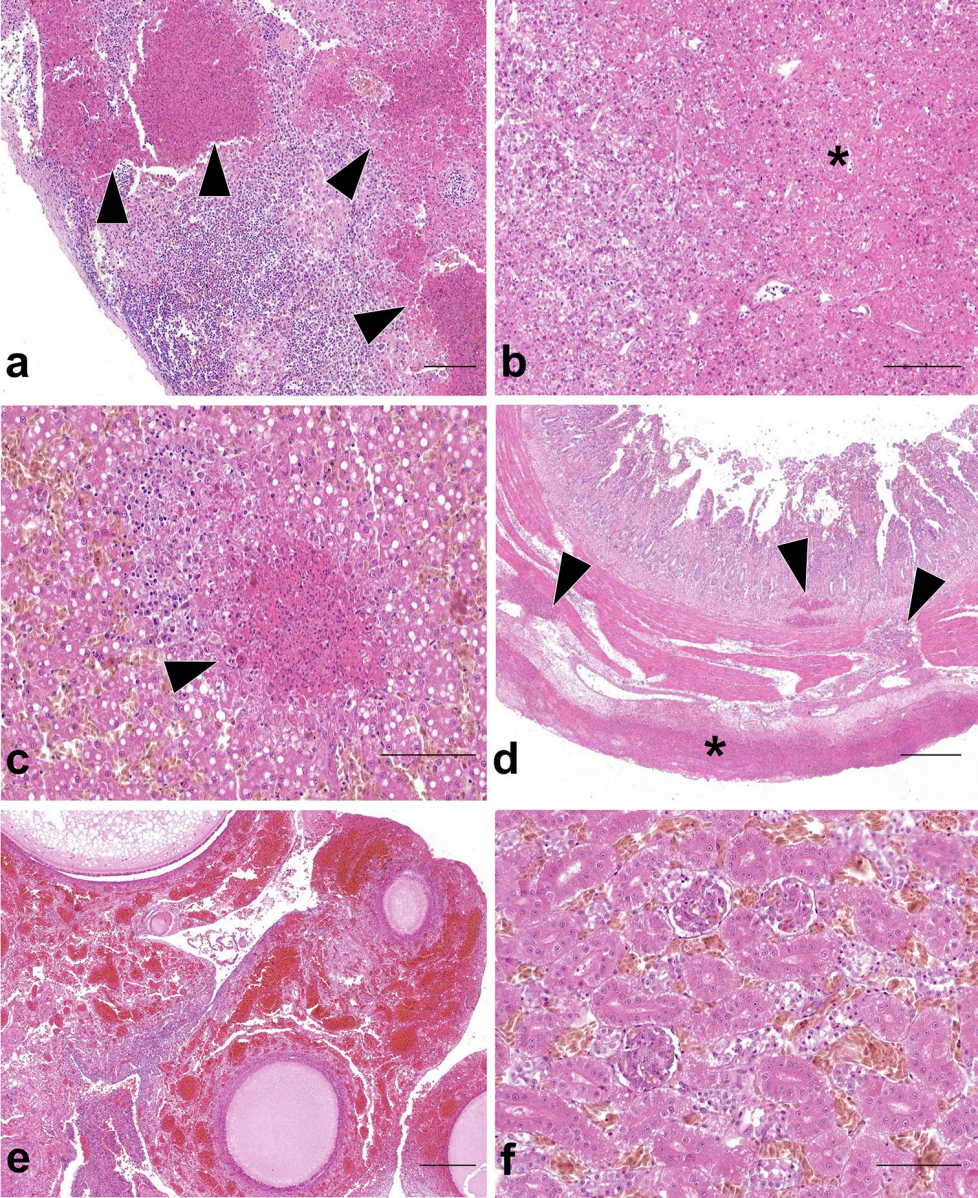
Histopathological lesions identified in African houbara bustards (*Chlamydotis undulata*) naturally-infected with H5N8 HPAIV. Tissue sections are stained with hematoxylin and eosin stain (H&E). Necrotic-inflammatory changes, ranging from multifocal to diffuse (arrowheads and asterisks) are present in spleen (**a**) pancreas (**b**) liver (**c**) and intestine (**d**). Generalized congestion predominated in ovary (**e**) and kidney (**f**). Scale bars: 100 μm (**a**–**c**, **f**) and 300 μm (**d**, **e**).

Results are summarized in Table 1. The spleen revealed severe necrotizing vasculitis with fibrinoid necrosis (4/4) and concurrent fibrino-heterophilic inflammation (3/4) (Fig. 2a). The pancreas exhibited mild to severe, parenchymal degeneration and lytic necrosis, with no to minimal mononuclear inflammation (5/5) (Fig. 2b). Hepatic lesions included diffuse macrovesicular steatosis (2/3), mild to moderate, multifocal lytic necrosis, with no to minimal mononuclear inflammation (3/3) and occasional fibrino-heterophilic exudation (1/3) (Fig. 2c). In the small intestine, the serosa was covered with large amounts of fibrino-heterophilic exudate, extending to the mesentery (1/2) (Fig. 2d). Several necrotic areas were scattered in the mucosa and submucosa, extending to the tunica muscularis (1/2). The tunica muscularis was also markedly expanded by lymphoplasmacells, occasionally organized in follicular structures exhibiting necrotic changes (1/2). Rare ulcers, were identified in the mucosa of ceca and colon-rectum (1/2). Moderate to marked generalized congestion was the only remarkable finding in the urogenital system (1/1) (Fig. 2e–f) and respiratory tract (3/3), while no lesions were observed in gizzard and heart sections (1/1).

**Table 1:** Clinico-pathological findings and viral tissue distribution by immunohistochemistry (IHC) and RNAscope in situ hybridization (ISH) in African houbara bustards (*Chlamydotis undulata*) naturally-infected with H5N8 HPAIV (n = 6).

Bird	Findings	Liver	Pancreas	Spleen	Gizzard	Intestine	Kidney	Gonad	Trachea	Lung	Heart
D1	Gross	Yes	Yes	Yes	No	Yes	Yes	Yes	No	Yes	Yes
	HE	+	+	na	**−**	**−**	**−**	**−**	**−**	**−**	na
	IHC	+++	+++	na	+	++	+++	+++	+	+++	na
	ISH	+++	+++	na	++	++	+++	+++	+	+++	na
S2	Gross	Yes	Yes	Yes	Yes	Yes	Yes	No	No	Yes	No
	HE	++	+++	+++	na	+	na	na	**−**	**−**	**−**
	IHC	+	+ +	+ +	na	+	na	na	+	+	**−**
	ISH	+	+++	+++	na	++	na	na	+	+	**−**
S3	Gross	Yes	Yes	Yes	No	No	Yes	No	Yes	No	No
	HE	+	++	+++	na	**−**	na	na	**−**	**−**	na
	IHC	++	+++	+++	na	+	na	na	+++	+	na
	ISH	++	+++	+++	na	++	na	na	+++	+	na
D4	Gross	Yes	Yes	Yes	No	Yes	Yes	No	Yes	Yes	No
	HE	na	+++	na	na	na	na	na	na	na	na
	IHC	na	+++	na	na	na	na	na	na	na	na
	ISH	na	+++	na	na	na	na	na	na	na	na
D5	Gross	Yes	Yes	Yes	No	No	Yes	No	Yes	No	No
	HE	na	na	+++	na	na	na	na	na	na	na
	IHC	na	na	+++	na	na	na	na	na	na	na
	ISH	na	na	+++	na	na	na	na	na	na	na
D6	Gross	Yes	Yes	Yes	No	Yes	Yes	No	Yes	No	No
	HE	na	++	+++	na	+++	na	na	na	na	na
	IHC	na	++	+++	na	+++	na	na	na	na	na
	ISH	na	++	+++	na	+++	na	na	na	na	na

D: found dead; S = symptomatic 24–48 h prior to the *exitus*; Gross: presence (yes) or absence (no) of lesions at the necropsy exam; HE: necrotic-inflammatory changes identified in histopathological slides routinely stained with hematoxilin and eosin, scored as −, +, ++, +++*; IHC: positivity for IAV antigen (nucleoprotein) scored as −, +, ++, +++*; ISH: positivity for AIV RNA (matrix gene) scored as −, +, ++, +++*; na: not available; *Semi-quantitative scoring according to Landmann^26^.

### In situ detection of avian influenza virus

**Figure 3:**
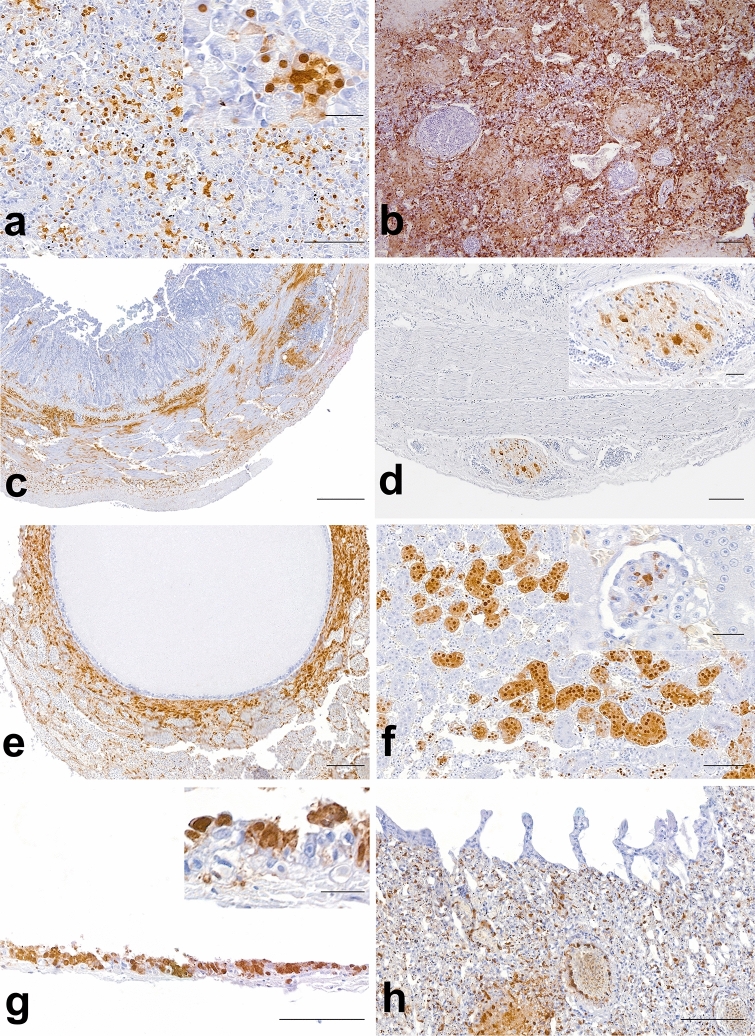
Viral antigen tissue distribution in African houbara bustards (*Chlamydotis undulata*) naturally-infected with H5N8 HPAIV. Tissue sections are stained for IAV nucleoprotein by immunohistochemistry. Multifocal immunolabelling of pancreatic acinar cells (**a**); widespread positivity of white and red pulp and necrotic foci in the spleen (**b**); multifocal transmural positivity of the intestine (**c**), including neuronal cells of the myenteric plexus (insert) (**d**); positive endothelial and mesenchymal cells in the ovary (**e**); multifocal to coalescing immunolabelling of the renal tubular epithelium and occasional glomeruli (insert) (**f**); diffuse positivity of the tracheal respiratory epithelium (insert) (**g**); multifocal positivity of the pulmonary capillary bed and endothelial cells lining the interlobular blood vessels (**h**). Scale bars: 100 μm (**a**, **b**, **d**–**h**), 300 μm (**c**) and 20 μm (inserts **a**, **d**, **f**, **g**).

**Figure 4:**
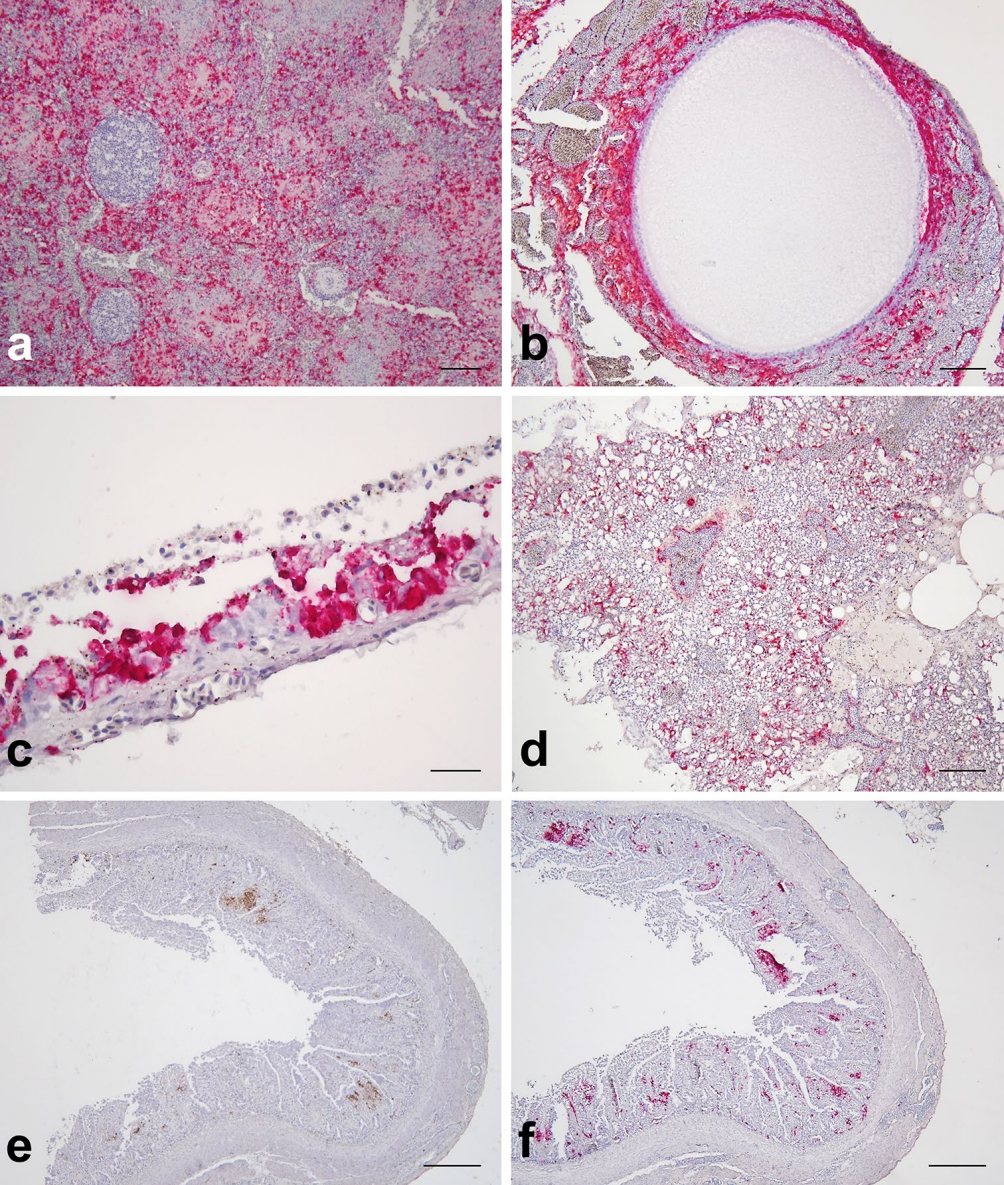
Viral RNA tissue distribution in African houbara bustards (*Chlamydotis undulata*) naturally-infected with H5N8 HPAIV. The first two rows include tissue sections stained for AIV matrix gene (M gene) RNA by RNAscope in situ hybridization (ISH). The third row includes serial intestinal sections stained for IAV nucleoprotein by immunohistochemistry (IHC) and M gene RNA by RNAscope ISH. Widespread positivity of splenic red and white pulp (**a**), mesenchymal and endothelial cells in the ovary (**b**), tracheal respiratory epithelium (**c**) and pulmonary capillary bed and blood vessels in the interparabronchial septa (**d**). Compared to IHC (**e**), RNAscope ISH revealed additional positive areas in the intestinal lamina propria (**f**). Scale bars: 100 μm (**a**, **b**, **d**), 300 μm (**e**–**f**) and 20 μm (**c**).

Results of viral tissue distribution and immunoreactivity are summarized in Tables 1, and 2. Viral antigens and RNAs were detected as intranuclear and intracytoplasmic staining in all tissues examined, except for the heart. For IHC, positive immunostaining was identified in liver sections within the hepatocytes (3/3), endothelial cells (3/3), cholangiocytes (2/3), periportal mononuclear leukocytes (2/3), stromal cells and mesothelial cells lining the Glisson’s capsule (1/3). In the pancreas, intact or necrotic acinar cells (5/5) followed by Langerhans’ islets cells and ductal epithelial cells (2/5) and occasional endothelial cells (4/5) appeared immunoreactive (Fig. 3a). Splenic sections showed multifocal to widespread positivity within the necrotic and fibrino-heterophilic foci (4/4), endothelial cells in both arterioles and penicillar capillaries (4/4), mononuclear leukocytes located in the white and red pulp (4/4), germinal centers (2/4), capsular smooth muscle cells (2/4) and ellipsoidal reticular cells (1/4) (Fig. 3b). In the intestine, viral antigen detection ranged from multifocal positive areas scattered within the lamina propria and neuronal cells of the myenteric plexus (3/4) to widespread immunoreactivity involving several compartments (1/4). In this case, positivity was recorded in smooth muscles cells in the lamina propria, muscularis mucosae and tunica muscularis, neuronal cells in the myenteric plexus, necrotic-inflammatory areas, as well as mononuclear leukocytes, endothelial cells and enterocytes (Fig. 3c–d). In the gizzard, the myenteric plexus was also positive, together with occasional endothelial cells in the lamina propria, and the interstitium of submucosa and tunica muscularis (1/1). In the ovary, endothelial and stromal cells of the vascular zone, followed by thecal cells and mesothelial cells, were highly positive (1/1) (Fig. 3e). Renal epithelial cells, in both cortical regions and medullary tracts, revealed large amounts of viral NP, while occasional positive cells were detected within glomerular tufts and interstitial blood vessels (1/1) (Fig. 3f). Viral antigen was also present in the ciliated epithelium of trachea (3/3) and secondary bronchi (1/3), tracheal intraluminal debris (1/3) pulmonary capillary bed (3/3) and endothelium of blood vessels in the interparabronchial septa (3/3) (Fig. 3g–h). Positive detection of viral RNA (M gene) by RNAscope ISH exhibited a tissue distribution equivalent to IHC (Fig. 4a–d). Additional positive areas were identified in gizzard (1/1) (mucosal epithelium and endothelial cells in the lamina propria) intestine (3/4) (mesothelial cells, mucosal epithelium, endothelial cells in the lamina propria) pancreas (1/5) (ductal epithelium and necrotic foci) and liver (2/3) (endothelial cells) (Fig. 4e–f).

**Table 2:** Endothelial viral detection by immunohistochemistry (IHC) and RNAscope in situ hybridization (ISH) in African houbara bustards (*Chlamydotis undulata*) naturally-infected with H5N8 HPAIV (n = 6).

Bird	Findings	Liver	Pancreas	Spleen	Gizzard	Intestine	Kidney	Gonad	Trachea	Lung	Heart
D1	IHC	+	+	na	+	++	++	+++	+	+++	na
	ISH	+	+	na	++	++	++	+++	+	+++	na
S2	IHC	−	−	+	na	−	na	na	+	++	−
	ISH	+		+	na	+	na	na	+	++	−
S3	IHC	−	+	++	na	−	na	na	+	++	na
	ISH	+	+	++	na	+	na	na	+	++	na
D4	IHC	na	+	na	na	na	na	na	na	na	na
	ISH	na	+	na	na	na	na	na	na	na	na
D5	IHC	na	na	+	na	na	na	na	na	na	na
	ISH	na	na	+	na	na	na	na	na	na	na
D6	IHC	na	+	+++	na	++	na	na	na	na	na
	ISH	na	+	+++	na	+++	na	na	na	na	na

D: found dead; S = symptomatic 24–48 h prior to the *exitus*; IHC: endothelial positivity for IAV antigen (nucleoprotein) scored as −, +, ++, +++* ISH: endothelial positivity for AIV RNA (matrix gene) scored as −, +, ++, +++*; na: not available; *Semi-quantitative scoring according to Landmann^26^.

### Molecular analysis on FFPE tissue blocks

Between 17.2 and 55.5 ng/µl of RNA (average 30.07 ng/µl) were obtained from 4 selected FFPE blocks. RNA integrity number (RIN) ranged from 5.5 to 7 (average 6.4), while DV200 score varied from 54.62 to 68.08 (average 60.59). AIV H5 was successfully detected by RT-qPCR in all 4 FFPE blocks, with a cycle threshold (Ct) value ranging between 21.23 and 25.05 (average 23.34). Partial sequencing of HA and NA highlighted a cleavage site consistent with HPAI and confirmed the involvement of N8 subtype, respectively (Accessions OR699288 and OR699289).

## Discussion

We provided the first comprehensive characterization of H5N8 HPAI natural infection in captive African houbara bustards, enriching available literature on the pathology of HPAI in an endangered avian species. A single report described an outbreak of H5N1 HPAI in Asian houbara bustards (*Chlamydotis maqueenii*) and falcons, exhibiting neurological signs and diarrhea^13^. The virus was isolated and molecularly characterized but, besides clinical signs, pathological findings were not provided and viral tissue distribution was not assessed. In another study, necrotizing pancreatitis was mentioned as a hallmark lesion in houbara bustards infected with H7N1 and H5N1 HPAI viruses but no additional details were provided^30^.

The birds included in our study exhibited hyperacute death, or presented with listlessness between 24 and 48 h prior to the *exitus*. Spleen, liver and pancreas were more commonly affected at necropsy, showing various degrees of degenerative changes, further confirmed at histopathology. Spleen sections also revealed marked fibrinoid necrosis, consistent with vasculitis. IHC and RNAscope ISH successfully highlighted the presence of viral antigen and RNA in the vast majority of the tissues examined. However, compared to IHC, RNAscope ISH appeared to be less affected by autolytic changes, and more sensitive. Positive detection was specifically recorded and consistent with multi-visceral, parenchymal and endothelial cells involvement, supporting a systemic infection and explaining the rapid course of the disease.

Interestingly, endothelial immunolabelling varied, in terms of frequency and severity, among affected bustards. A possible explanation for this finding is the fact that, in some individuals, death may have occurred before extensive viral replication within the vascular endothelium^20^. Compared to Anseriformes, with the exception of the black swan (*Cygnus atratus*), systemic endotheliotropism with necrotizing vasculitis and hyperacute/acute forms, are considered hallmarks of HPAI virus infection in domestic Galliformes^20,31^. In chickens, endothelial cells are reported to be more permissive to HPAI virus infection, compared to ducks, due to a lower antiviral response^31^. An acute course of disease (short mean death time) in chickens is also associated with a reduced viral excretion time. On the other end, ducks have a longer mean death time and shed more virus over the course of the infection^32,33^. Overall, since several factors may have been involved, including species affected, host genetic polymorphism, course of the disease and concurrent infections, additional studies are needed to properly define the role played by endothelial cells in HPAI infection in bustards^34^.

In terms of tissue distribution, large amounts of AIV were detected in the urogenital tract of bustards, despite the absence of significant lesions, except for generalized congestion. Similarly, H5N1 HPAI was able to replicate in the renal tubular epithelium of both naturally and experimentally-infected waterfowl, without inducing significant changes^35,36^. However, renal lesions with concurrent positive immunolabelling were reported in wild Baikal teals (*Anas Formosa*), bean geese (*Anser fabalis*), and whooper swans (*Cygnus Cygnus*) and backyard chickens naturally-infected with H5N8 HPAI^37,38^. The involvement of the reproductive tract was mentioned in different avian species exposed to several HPAI subtypes. H5N8, H5N2 and H5N1 viruses were detected by RT-qPCR and/or IHC in the oviduct, eggshell and internal egg contents of experimentally-infected white leghorn chickens (*Gallus gallus domesticus*)^39,40^. IHC revealed IAV antigen in the ovarian stroma of commercial meat-type turkeys (*Meleagris gallopavo*) naturally-infected with H5N8 HPAI and in several tissues, including the reproductive tract, of Passeriformes and budgerigars (*Melopsittacus undulatus*) experimentally-infected with H5N1 HPAI^41,42^.

A limitation of this study was the lack of systematic tissue sampling, regardless the presence of specific clinical signs and/or macroscopic lesions. Non-suppurative, necrotizing encephalitis and myocarditis have been commonly described in a variety of commercial, captive and wild birds, naturally and experimentally-infected with H5N8 HPAI^14,15,24,42–44^. In our case, the presence of cerebral lesions couldn’t be assessed because the nervous system was not collected due to the lack of neurological signs. However, immunoreactivity of the myenteric plexus in the gastrointestinal tract, confirmed by both IHC and RNAscope ISH, still supported a neuronal tropism. Cardiac sections appeared unremarkable although, false negative results may have originated from a non-uniform distribution of lesions and from the fact that only 1/6 birds was sampled.

In birds infected with HPAI viral shedding can occur through the respiratory, digestive and urogenital tract^20^. In addition, due to systemic infection, large amounts of virus are present within a variety of tissues, including musculoskeletal and integumentary systems, making scavenging birds particularly at risk of being exposed following the consumption of infected carcasses^13–15,20,45,46^. Non-respiratory particles, such as dust originating from infected poultry houses, have also been recognized as an important source of infection^47–49^. In our case, two nests belonging to an unidentified waterfowl species, were discovered nearby the first positive pen, making the exposure to infectious aerosol, fecal particles as well as infected feathers and dust, a plausible event.

Molecular analysis conducted on FFPE tissue blocks, further confirmed the involvement of H5N8 HPAI in affected bustards. Nucleic acids fragmentation, resulting from formalin-fixation makes short-read sequencing technologies more suitable for FFPE tissues. However, we chose to assess the feasibility of the Oxford Nanopore MinION, a long-read technology, since it can generate real-time results, is economically affordable and portable^50^. To the author’s best knowledge, this is the first time nanopore sequencing has been used on FFPE samples for veterinary viral investigation. FFPE blocks represent practical samples that can be stored at room temperature and can be easily shipped. They are an invaluable resource of information for retrospective studies, and diagnostic investigation of poorly characterized conditions and unsolved archived cases, which is particularly interesting for exotic and endangered species with a limited availability of samples. In addition, preserved tissue architecture and histomorphological features allow target and custom-designed testing, increasing sensitivity. An example is provided by the molecular identification of *Toxocara cati* in FFPE blocks obtained from brown kiwis (*Apteryx mantelli*) with a histopathological diagnosis of visceral and neural *larva migrans,* between 2004 and 2017^51^. Nevertheless, Gaide et al*.*^28^ successfully detected AIVs by RNAscope ISH and IHC in FFPE blocks stored at room temperature between 2009 and 2022, with no detrimental effects on final results.

In conclusion, our results show that natural infection with H5N8 HPAI virus in African houbara bustards is characterized by hyperacute/acute forms exhibiting pantropism, as well as endotheliotropism and neurotropism. Significant pathological indicators to be considered at necropsy include necrotizing splenitis, pancreatitis and hepatitis and fibrinous peritonitis. FFPE samples represent a valuable source of nucleic acids for in situ detection and molecular analysis of emerging and re-emerging avian pathogens in poorly characterized species.

## Data Availability

The datasets generated and/or analyzed during the current study are available in the GenBank repository, Accession Numbers OR699288 and OR699289.
